# Determinants of respectful maternity care among women who gave childbirth in Southern Ethiopia

**DOI:** 10.1186/s12913-024-10813-7

**Published:** 2024-04-10

**Authors:** Dawit Utalo, Eskinder Israel, Tsegaye Lolaso Lenjebo, Amdehiwot Aynalem, Tadele Dana Darebo

**Affiliations:** 1 Departement of Public Health, Consortium Project at Women Empowerment-Amref Health Africa, Wolaita Sodo, Ethiopia; 2 School of Public Health, College of Health Science and Medicine, Wolaita Sodo University, Wolaita Sodo, Ethiopia; 3 School of Nursing, College of Medicine and Health Science, Hawassa University, Hawassa, Ethiopia

**Keywords:** RMC, Respectful, Maternity, Intrapartum, Determinants, Southern Ethiopia

## Abstract

**Background:**

Having a good provision of respectful maternity care (RMC) to a woman who gives childbirth is a crucial component of maternal health care to result in positive maternal and neonatal outcomes. Disrespect and lack of women-centered care in birth discourage a woman from seeking healthcare during childbirth contributing to poor healthcare-seeking behaviour and dissatisfaction with the maternity service. The current study aimed to assess key determinants of RMC during childbirth at selected public health facilities of the Gofa zone, Southern Ethiopia.

**Methods:**

A cross-sectional study design was conducted from March to April 2021 among 390 women who gave birth in eight randomly selected public health facilities of Gofa zone, Southern Ethiopia. The level of RMC was measured using structured exit interview items. A structured-interviewer-administered questionnaire was used to collect data and then entered into Epi-data version 4.6 and exported to SPSS version 25 for further analysis. Bivariate and multivariate logistic regression analyses were used to identify determinants of RMC among women.

**Results:**

A total of 390 women responded to the exit interview making a response rate of 100%. The mean (± SD) age of the 390 women was 27.9 (± 4.85) years. The overall prevalence of women who received RMC was 40.5%, 95% CI (36-45%). Two hundred and ninety-seven (76.2%; *n* = 297/390) women had antenatal care (ANC) attendance in the index pregnancy. A woman who had planned pregnancy (AOR = 1.72, CI: 1.04, 2.85), planned to deliver in a health facility (AOR = 1.68, CI: 1.00, 2.81), presence of familial support (AOR = 2.04, CI: 1.20, 3.48), and had information about service availability (AOR = 4.44, CI: 2.09, 9.42) were associated with RMC among women.

**Conclusion:**

The provision of respectful maternity care in the study area was low when compared with local studies. Planned pregnancy, plan to deliver in a health facility, family support, and presence of information about service availability were factors associated with RMC among women. More attention should be given to training and supportive supervision of health care professionals on respectful maternity care and its standards to increase service uptake and make service more women-centred.

## Background

The World Health Organization (WHO) defines respectful maternity care (RMC) as comprehensive care given to women in a manner that ensures respect for their sexual and reproductive health (SRH) and their fundamental rights [1]. It is a care delivered to a women with all their full dignity, and privacy as well as in a confidential manner that guarantees their right to seek and obtain care without any harm or mistreatment [2].

Globally, nearly 134 million women need protection from harm and mistreatment and need a due assistance during childbirth [3]. Sub-Saharan African countries, where more than three-fourths of maternal death happens, exhibit poor RMC when compared to other countries [4]. Various studies from different countries reported variation in rate such as Nigeria and Nepal reported the overall rate of RMC was 69.9% and 84.7% respectively [5, 6]. Non-consented care, lack of privacy, and non-confidential care were reported as the common disrespect and abuse faced by the woman in their childbirth. Good provision of RMC to a woman who gives childbirth is an important component of maternal health care to result in positive maternal and neonatal outcomes [7]. Disrespect and lack of women-centered care in childbirth discourage a woman from seeking healthcare and contributing to poor healthcare-seeking behaviour and dissatisfaction with the maternity service [3]. Different studies in the field indicated that RMC is important not only to promote the uptake of facility-based childbirth but also to improve the clinical childbirth outcomes and to reduce potential fetomaternal complications (FMC) associated with childbirth [8]. The absence of a champion during childbirth, substandard maternity housing, long waiting times to receive care, and disrespect and abuse during birth were factors that contributed to the underutilization of facility-based childbirth [9].

Despite having good maternal and child health (MCH) program improvement in the past five years in Ethiopia, a reduction in maternal mortality ratio (MMR) would not have been further recorded. Studies conducted in public hospitals of Eastern and Western Ethiopia indicated the RMC rate of 38.4% and 35.8% respectively [10, 11]. Demography and Health Survey (DHS) report of 2016 in Ethiopia showed MMR of 412/100,000 live births (LB) [12] which is far from the target to be achieved by Sustainable Development Goal (SDG) by 2030 [13]. Additionally, report from the Ethiopian Mini DHS 2019 showed only 48% of the women gave their childbirth in the health facilities [14]. To tackle this, Federal Ministry of Health (FMOH) planned to increase the number of births attended by a skilled birth attendant (SBA) from 15% in 2014 to 90% by 2020 through Health Sector Transformational Plan (HSTP) IV [15]. It also launched a new compassionate and respectful maternity care (CRC) program to understand the client’s context and their perspective, to meet their needs and guide them for evidence based decision-making [9, 12]. Further efforts were also done to establish maternal waiting rooms, making labor and delivery service free of charge and aimed at creating pregnant women’s conferences [16].

Assessing RMC in childbirth is so important to design, monitor and evaluate various interventions to promote best practice during childbirth, particularly in resource limited setting including Ethiopia. Despite this, the data is limited in the study area. Therefore, this study is aimed to identify determinants of intrapartum RMC among women agreeing to the context of the community in the study area, particularly in selected public hospitals of Gofa Zone, Southern Ethiopia, 2021.

## Methods

### Study design, period, and setting

A cross-sectional study design was employed from March to April 2021 in a selected public hospital in Gofa Zone, Southern Ethiopia, located 514 km away from Addis Ababa, capital of Ethiopia. Nearly 130,548 women are currently utilizing maternity services from Gofa zone health facilities. The zonal structure has two hospitals (one general and one district), 14 health centers, and 175 health posts and has more than 150 health care providers serving in the maternal and child health (MCH) unit [17].

### Population

All women who gave birth at the public health facilities of the Gofa zone were regarded as the source population and women who gave birth in selected public health facilities of the Gofa zone and lived for at least six months in the study area were the study population. All selected women who gave birth in selected public health facilities and lived at least six months in the study area were included in the study.

All selected women who were referred to other health facilities and were critically ill at the time of data collection were excluded from the study.

### Sample size determination and sampling technique

The sample size was calculated with a single population proportion formula using

$$ \text{n}-\frac{{\left({\text{z}}_{{\alpha }/2}\right)}^{2}\times \text{p}(1-\text{p})}{{\text{d}}^{2}},$$

Z_1−α/2_—significance level at α = 0.05 (standard normal variable at 95% confidence level = 1.96).

d—Expected margin of error usually 5%.

P—the expected proportion of RMC among women who gave birth in west Oromia public health facilities in Ethiopia, is 35.8% [11] thus yielding 354 and then adding 10% of the non-response rate, the final sample size obtained from the first objective was 390. We also calculated the sample size for the second objective using Epi info version 7 to calculate for determinants (Table 1). However, a small sample size was found and we took the largest sample size of the first objectives (*n* = 390).

**Table 1:** The table shows sample size calculation for the determinants based on factors reviewed from previous studies using open Epi version7 for cross-sectional studies

Factors	Percent of unexposed with outcome	Two-sided confidence level	AOR	Power	Zα/2	The ratio of unexposed to exposed	Sample size	Ref.no
Place of delivery preference	35	95%	2.3	80	1.96	1	206	[18]
Duration of stay at health facilities	28.5	95%	2.1	80	1.96	1	272	[9]
Place of residence	22	95%	3.3	80	1.96	1	118	[19]

**Fig. 1:**
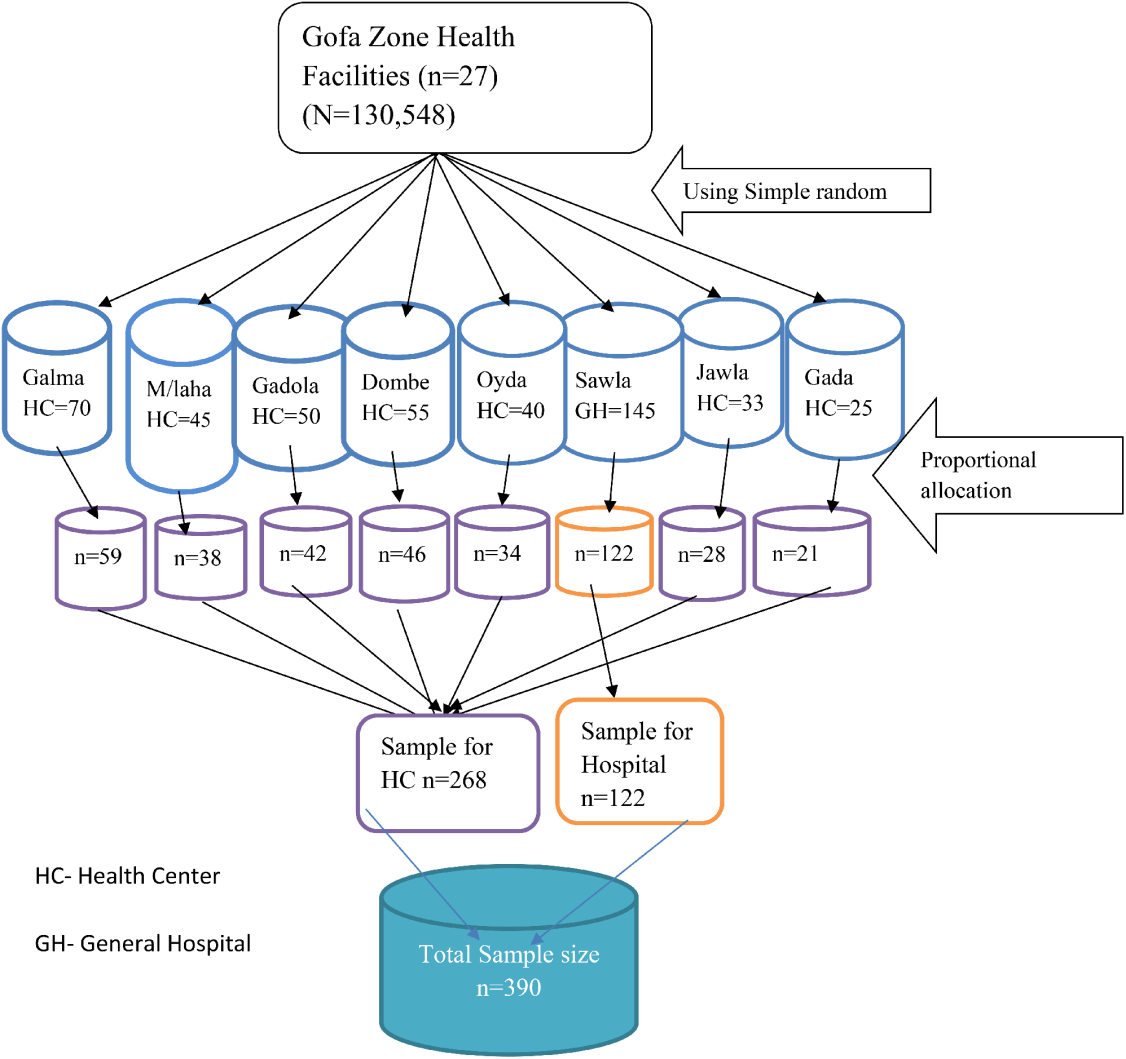
A flow chart showing a sampling procedure for Respectful Maternity Care among women who gave birth in Gofa Zone, Southern Ethiopia, 2021

Eight health facilities (one general hospital (GH) and seven health center (HC)) were selected randomly from the total health facilities found in the Gofa zone. Then, a proportional allocation of the sample size to the number of women who gave childbirth based on the previous month’s data (*n* = 432) was done to obtain the required sample size. Finally, a simple random sampling technique was employed to select each woman from each health institution (Fig. 1).

### Operational definitions

**Respectful maternity care (RMC)** is a composite variable measured using structured exit interview items that describe the desired behavior of the healthcare provider. The items contain eight questions that describe the desired behavior of the healthcare provider. The desirable provider behaviours included receiving and greeting women, explaining each step of the examination, encouraging women to ask any questions, responding to women and their companions politely, explaining to women what will happen in childbirth, encouraging them to walk and change position as preferred, ensuring and helping them to eat light food and lastly, asking women their preferred position of giving childbirth.

The outcome variable was the sum of eight equally weighted RMC practice tools that ranged from 0 to 8 (39). Women who answered yes to four (4) and above RMC assessing questions from eight questions were considered as women who received RMC.

### Data collection tools, procedure

Data were initially adapted from WHO, USAID, and previous studies [4, 16, 19, 20]. The questionnaire consists of socio-demographic characteristics, obstetric-related characteristics, and provider-related characteristics. The questionnaire was primarily prepared in English and then translated into Amharic and retranslated back into the English language to check for possible consistency. Seven BSc midwives who work in other health facilities collected data and were supervised by two MPH holders who had good experience in collecting quantitative data.

### Data management and analysis

Pre-testing of the data was conducted among 5% (*n* = 29) of the total sample size in one of the unselected public health facilities before actual data collection to ensure quality. After the pre-test, clarifications and corrections were made to the questionnaire to ensure the consistency of the tool. The English version questionnaire was translated into Amharic and then to Gofigna by the language expert and then back to English to maintain its consistency. During data collection, the investigator and supervisors checked the collected data every day for completeness and consistency throughout the data collection period. Data were initially checked for completeness and entered into EPi-data version 4.6 then exported to SPSS version 25 for further statistical analysis. Bivariate and multivariable logistics regression analysis was conducted to identify an association between the dependent and independent variables. Variables with *p* < 0.25 on the bivariate analysis were entered into multivariable logistic analysis and those variables with *p* < 0.05 in the final model were taken as statistically significant.

## Results

### Socio-demographic characteristics of the women

A total of 390 women responded to the exit interview making a response rate of 100%. The mean age of women was 27.9 (± 4.85) years. The majority of the women, 356 (91.3%) were married. Regarding women’s occupational status, 229 (58.7%) were housewives, and more than one-third, 265 (67.9%) were from rural. Nearly one-third (31.5%) of the women attended their primary level education (Table 2).

**Table 2:** Socio-demographic characteristics of the women at selected public health facilities of Gofa zone, Southern Ethiopia, 2021

Variables	Categories (*n* = 390)	n (%)
Residence	Urban	125 (32.1)
	Rural	265 (67.9)
Occupation	Housewife	229 (58.7)
	Government employee	90 (23.1)
	Merchant	44 (11.3)
	Private employee	13 (3.3)
	Others	14 (3.6)
Marital status	Married	356 (91.3)
	Divorced	23 (5.9)
	Widowed	7 (1.8)
	Other	4 (1)
Educational status	No formal education	112 (28.7)
	Primary Education (1–8)	123 (31.5)
	Secondary education (9–12)	64 (16.4)
	College and above	91(23.3)

### Obstetric-related characteristics of women

More than one-fourth (27.2) of the women were para I. One hundred and ninety-two (49.2% ) women had a previous history of facility childbirth, and 297 (76.2%) women had antenatal care (ANC) follow-up. 180 (46.2%) women had 6–12 h of median waiting time in the health facility during facility-based birth.

More than one-third (67.7%) of the woman gave birth with spontaneous vaginal delivery (SVD) and 82 (21%; *n* = 82/390) women delivered with the aid of episiotomy. Nearly half (48.7%) of women stayed in the health facility before their labor commenced (Table 3).

**Table 3:** Obstetric-related characteristics of women at selected health facilities of Gofa zone, Southern Ethiopia, 2021

Variables	Categories (*N* = 390)	n (%)
Parity	One	106 (27.2)
	Two	113 (29.0)
	Three	75 (19.2)
	Four	57 (14.6)
	Five and above	39 (10)
History of health facility birth	Yes	192 (49.2)
	No	198 (50.8)
Frequency of facility-based childbirth	One	153 (39.2)
	Two	129 (33.1)
	Three	83 (21.3)
	Four and above	25 (6.4)
Planned current pregnancy	Yes	200 (51.3)
	No	190 (48.7)
ANC follow-up during the index pregnancy	Yes	231 (59.2)
	No	159 (40.8)
Place of ANC follow-up	Health Center	297 (76.2)
	Hospitals	60 (15.4)
	Private health facilities	1 (0.3)
	Health posts	32 (8.2)
Duration of labor	Less than 6 h	69 (17.7)
	6–12 h	180 (46.2)
	13–24 h	122 (31.3)
	Greater than 24 h	19 (4.9)
Mode of delivery	Spontaneous vaginal delivery	264 (67.7)
	Delivery with episiotomy	82 (21)
	C-section delivery	30 (7.7)
	Instrumental delivery	14 (3.6)
Family support during childbirth	Yes	231 (59.2)
	No	159 (40.8)
Stayed at health facilities before labor started	Yes	190 (48.7)
	No	200 (51.3)
Number of days stayed at the facility before the start of labor	One day	308 (79)
	Two days	54 (13.8)
	Three days	23 (5.9)
	Four days and above	5 (1.3)
Postnatal care service	Yes	273 (70)
	No	117 (30)
ANC visit	One	108 (27.7)
	Two	43 (11)
	Three	79 (20.3)
	Four and above	160(41)

### Place of birth preference by women during the index pregnancy

**Fig. 2:**
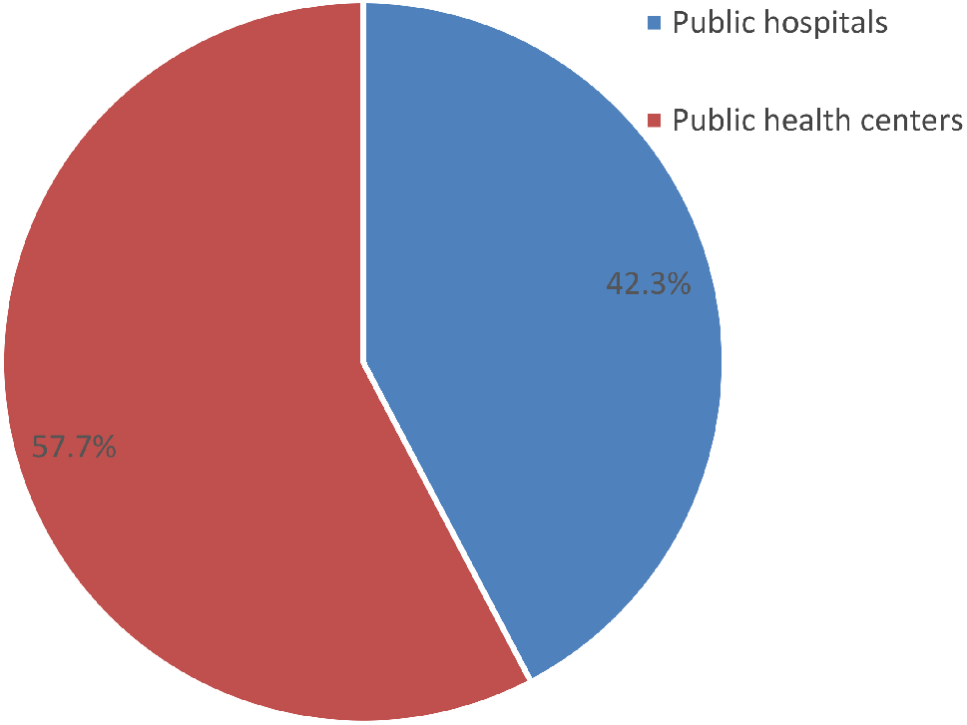
A figure showing a preference for place of childbirth by women during the index pregnancy in Southern Ethiopia, 2021

More than half, 225 (57.7%) women preferred to have at the health centres, and 165 (42.3%) women at the hospital (Fig. 2).

### Provider level-related characteristics

Women were asked to recall the number of healthcare providers who attended their childbirth. More than two healthcare providers attended 167 (42.8%) women during childbirth. More than one-third (68.7%) of the women were attended by female healthcare providers. About 262 (67.2%) women were counselled for birth preparedness and complications readiness plans during their childbirth. Nearly three-fourths of the women were also planned to deliver in the health facilities (Table 4).

**Table 4:** Provider level related characteristics at selected health facilities of Gofa zone, Southern Ethiopia, 2021

Variables	Categories (*N* = 390)	n (%)
Counselled for birth preparedness	Yes	262 (67.2)
	No	128 (32.8)
Plan to deliver in the health facility	Yes	291 (74.6)
	No	99 (25.4)
Attendants during childbirth (in number)	One	43 (11)
	Two	167 (42.8)
	Three	166 (42.6)
	Four and above	14 (3.6)

### Service delivery-related characteristics

**Fig. 3:**
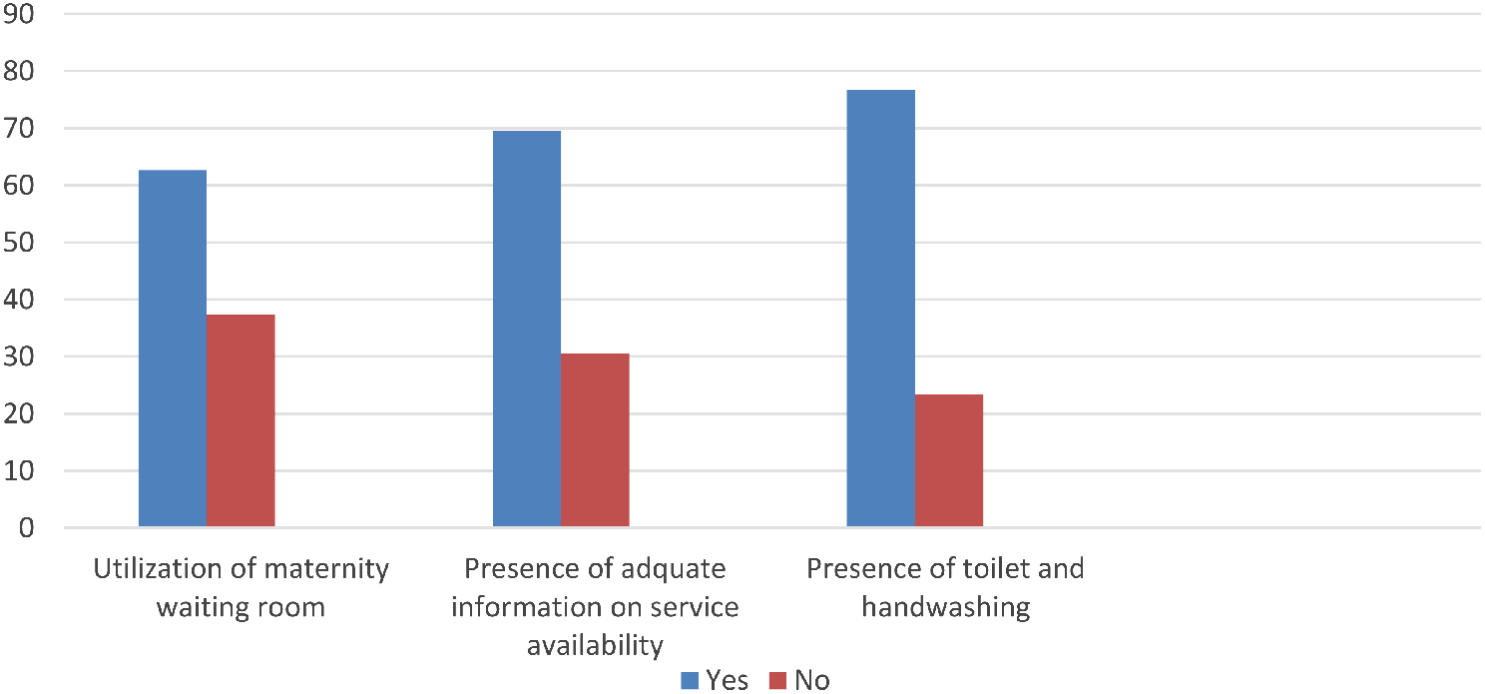
A figure showing service delivery level-related characteristics of women at selected public hospitals of Gofa zone, Southern Ethiopia, 2021

Two hundred and seventy-eight (71.3%) women’s privacy was protected during childbirth. More than one-third (69.5%) of the women had adequate information about the availability of services in their health facilities. Two hundred and ninety-nine (76.7%) women had toilet and hand washing services in their health facilities. Regarding maternity waiting room utilization, 244 (62.6%) women utilized it (Fig. 3).

### Overall status of RMC among women who gave birth

**Fig. 4:**
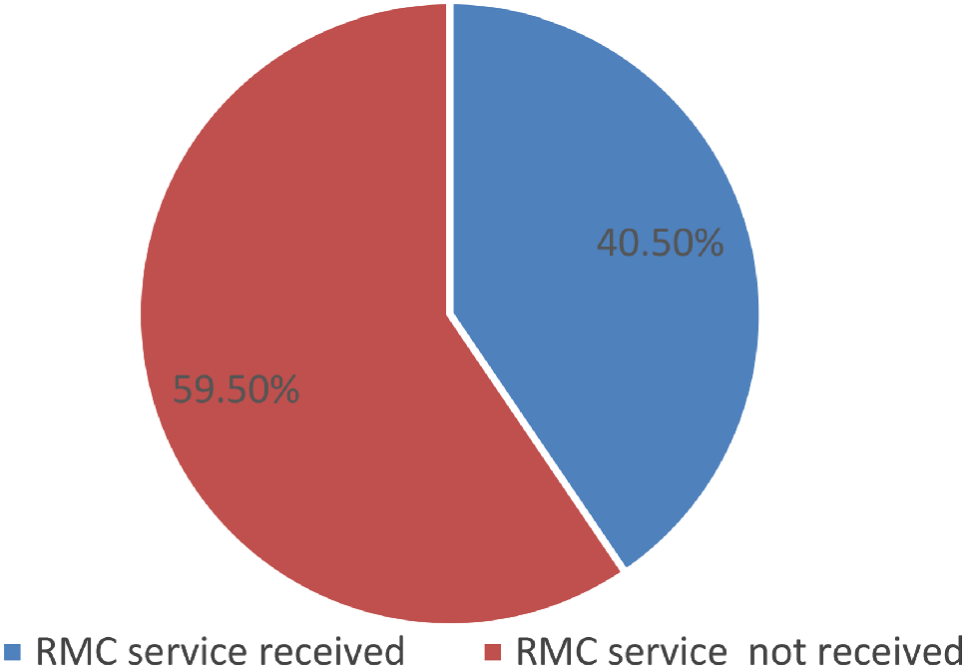
Figure showing the overall status of RMC among women who gave birth in Gofa Zone, southern Ethiopia, 2021

One hundred and eight (40.5%, 95%: CI-36-45%) women had respectful maternity care during facility-based birth (Fig. 4).

### Categories to measure RMC during facility-based birth

Two hundred and fifty-two (64.6%) women were welcomed by their healthcare providers and 208 (53.3%) women got informed consent during the birth procedure. Nearly half, 191(49.0%) women were encouraged to ask any questions and 214 (54.9%) women were encouraged to walk and change positions during birth. In addition to this, 174 (44.6%) women were encouraged to take light foods and fluids. Nearly half, (50.5%) of women asked about their comfort position during birth and 235 (60.3%) women were allowed to give birth in their preferred positions (Table 5).

**Table 5:** Categories to measure RMC during facility-based birth at selected public health facilities of Gofa zone, Southern Ethiopia, 2021

Variables	Characteristics (*N* = 390)	n (%)
Women welcomed during childbirth	Yes	252 (64.6)
	No	138 (35.4)
Informed consent was given during childbirth	Yes	208 (53.3)
	No	182 (46.7)
Encouragement from health workers to ask any questions was done	Yes	191 (49)
	No	199 (51)
Health professionals respond to your questions politely	Yes	191 (49)
	No	199 (51)
Health professionals encourage to walk and position during childbirth	Yes	214 (54.9)
	No	176 (45.1)
Women encouraged to take light foods and fluids during childbirth	Yes	174 (44.6)
	No	216 (55.4)
Health professional asked women their position of delivery	Yes	197 (50.5)
	No	193 (49.5)
Health professionals allowed the position women preferred	Yes	235 (60.3)
	No	155 (39.7)

### Determinants of RMC among women who gave birth at Gofa Zone public health facilities, southern Ethiopia, 2021

Six variables with a p-value of ≤ 0.25 were selected and entered into the multivariate logistic regression analysis model. These are planned pregnancy, adequate information on service availability, counselling for birth preparedness, family support, a plan to deliver in a health facility, and an ANC visit.

In multivariate logistic regression women who had planned pregnancy, planned to deliver in the facility, had family support, and had adequate information on maternal service availability were associated with RMC.

Women who had planned pregnancies were more likely to get RMC as compared with women who had no planned pregnancy (AOR = 1.65, 95% CI; 1.00- 2.815). Women who planned to deliver in the health facility were more likely to get RMC than those who weren’t. In addition, women who had support from their family during childbirth were 2.04 times more likely to have RMC than women who had no support from their family (AOR = 2.04, 95% CI; 1.20, 3.47). Women who had information about the availability of maternity services in a health facility were 4.50 times more likely to get RMC than women who had no information (AOR = 4.50, 95%, CI; 2.28–8.87) (Table 6).

**Table 6:** Determinants of RMC among women who gave birth at selected health facilities of Gofa zone, Southern Ethiopia, 2021

Variables	Category	RMC		COR (95% CI)	AOR (95% CI)
		Yes	No		
Planned pregnancy	Yes	51	139	1	1
	No	107	93	0.31(0.20,0.48)*	1.65(1.00,2.81)**
Availability of adequate information	Yes	8	66	1	1
	No	150	166	0.13(0.06,0.28)*	4.50 (2.28,8.87)**
Counselled for birth preparedness	Yes	46	80	1	1
	No	112	150	0.75 (0.48,1.16)	1.55 (0.84,2.85)
Familial support	Yes	42	117	1	
	No	116	115	0.35 (0.23,0.55)*	2.04 (1.20,3.47)**
Plan to deliver in the health facility	Yes	19	80	1	1
	No	139	152	0.26 (0.15,0.45)*	1.53 (0.32,0.89)**
ANC Attendance	One	57	51	1	1
	Two	7	36	1.63 (0.99,2.67)	3.64 (0.49,27.09)
	Three	29	50	0.28 (0.12,0.68)*	3.71 (0.56,24.4)
	Four and above	65	95	0.85 (0.49,1.48)	2.64 (0.41,16.43)

* p- value < 0.001

** p- value < 0.005

## Discussion

The current study revealed the determinants of intrapartum RMC among women in selected public health facilities at Gofa Zone, Southern Ethiopia. In this study, less than half of women received RMC during facility-based birth, which was 40.5%, 95% CI (36-45%). This figure is slightly higher than the study conducted in Harar, Eastern Ethiopia which indicated that RMC among women who gave birth was 38.4% [10]. It’s also higher than the study conducted in western Ethiopia in which RMC during facility-based birth was 35.8% [11]. This variation might be due to differences in sociocultural status, the healthcare-seeking behavior of the women and differences in the study setting.

The finding from this study was lower than the study conducted in three regions of Ethiopia (Oromia, Southern, and Amhara) which showed women who received RMC was 66% during birth [19], and the study conducted in Northern Ethiopia which revealed RMC experienced was 57% [21]. It was also lower than the study conducted in Iran which reported RMC among those who gave birth was 62.5% [22]. This difference might be due to the variation in the provision of information to pregnant women who gave birth, the difference in the study setting, and the attitude of the health care providers towards RMC.

Women with planned pregnancies were more likely to get RMC than those women who had no planned pregnancy. This finding is consistent with the study from Eastern Ethiopia [10]. This might be due to the reason that women with planned pregnancies were more concerned about the outcomes of the pregnancy and they may have positive health-seeking behavior. Evidence showed that the desired pregnancy increases women’s level of gratification and recognizes the service in public health facilities [18].

Women who had support from family during birth were two times more likely to have RMC than women who had no support [18, 23]. This could be due to they may receive continuous emotional support from their husband and utilize maternity services in a better way. In line with this, it was indicated that the presence of family in the labor ward increases dignified and supportive care and is associated with better maternal and birth outcomes that further result in respectful maternity care [24, 25].

In addition, this study also indicated that women who planned to deliver in health facilities were more likely to get respectful and abuse-free care than women who had no plan to deliver in health facilities. This could be justified as when women plan to get delivery service at the health facilities, they start to plan for getting counselling and related maternity service from health care providers that further enhances and maintains good respectful and abusive free care [26, 27].

Women who had adequate information on maternity services were nearly five times more likely to receive RMC than women who had no information. This finding is in line with the study conducted in West Ethiopia which revealed 39.3% of women had the right information regarding services given in health facilities that helped them to get respectful care [11]. The possible justification for this could be women who had frequent visits to health facilities and discussed maternity issues were more likely to be familiar with service providers and this can significantly enhance RMC service [28, 29].

The study has some limitations. Firstly, since the study is cross-sectional, a causal association would not have been inferred. Secondly, as midwives collected data, selection bias might have occurred. Finally, we were unable to study the comparative analysis between different types of health post, and different communities which gives nice input to programmers and policy makers. We suggest that future studies should take these limitations into account to improve the robustness of studies on similar topics. Despite this, this study tried to determine key determinants of intrapartum respectful maternity care in the study area.

## Conclusion

The provision of respectful maternity care in the study area was low when compared with local studies. Planned pregnancy, plan to deliver in a health facility, family support, and presence of information about service availability were factors associated with RMC among women. More attention should be given to training and supportive supervision of health care professionals on respectful maternity care and its standards to increase service uptake and make service more women-centred.

## Data Availability

All data included in this manuscript can be accessed from the corresponding author upon request through the email address.

## Declarations

We also received informed consent from all women before conducting the actual study. All methods were also carried out in accordance with relevant guidelines and regulations or the declaration of Helsinki.

## Abbreviations

ANC: Antenatal Care
AOR: Adjusted Odds Ratio
CI: Confidence Interval
CRC: Compassionate Respectful Care
EDHS: Ethiopia Demographic Health Survey
FDRE: Federal Democratic Republic of Ethiopia
MCH: Maternal and Child Health
MMR: Maternal Mortality Ratio
RMC: Respectful Maternity Care
SDG: Sustainable Development Goals
SPSS: Statistical Package For Social Science
SRH: Sexual and Reproductive Health
UNICEF: United Nations Children’s Fund
WHO: World Health Organization
